# Genome-wide patterns of differentiation within and among U.S. commercial honey bee stocks

**DOI:** 10.1186/s12864-020-07111-x

**Published:** 2020-10-08

**Authors:** Perot Saelao, Michael Simone-Finstrom, Arian Avalos, Lelania Bilodeau, Robert Danka, Lilia de Guzman, Frank Rinkevich, Philip Tokarz

**Affiliations:** 1 USDA-ARS, Honey Bee Breeding, Genetics and Physiology Laboratory, Baton Rouge, LA 70820 USA; 2 Present Address: USDA-ARS Knipling-Bushland U.S. Livestock Arthropod Pests Research Unit, Kerrville, TX 78028 USA

**Keywords:** *Apis mellifera*, Pool-seq, F_ST_, Single nucleotide polymorphism, Genetic diversity, Stock identification

## Abstract

**Background:**

The population genetics of U.S. honey bee stocks remain poorly characterized despite the agricultural importance of *Apis mellifera* as the major crop pollinator. Commercial and research-based breeding programs have made significant improvements of favorable genetic traits (e.g. production and disease resistance). The variety of bees produced by artificial selection provides an opportunity to characterize the genetic diversity and regions of the genome undergoing selection in commonly managed stocks.

**Results:**

Pooled sequencing of eight honey bee stocks found strong genetic similarity among six of the stocks. Two stocks, Pol-line and Hilo, showed significant differentiation likely due to their intense and largely closed breeding for resistance to the parasitic *Varroa* mite. Few variants were identified as being specific to any one stock, indicating potential admixture among the sequenced stocks. Juxtaposing the underlying genetic variation of stocks selected for disease- and parasite-resistance behavior, we identified genes and candidate regions putatively associated with resistance regulated by hygienic behavior.

**Conclusion:**

This study provides important insights into the distinct genetic characteristics and population diversity of honey bee stocks used in the United States, and provides further evidence of high levels of admixture in commercially managed honey bee stocks. Furthermore, breeding efforts to enhance parasite resistance in honey bees may have created unique genetic profiles. Genomic regions of interest have been highlighted for potential future work related to developing genetic markers for selection of disease and parasite resistance traits. Due to the vast genomic similarities found among stocks in general, our findings suggest that additional data regarding gene expression, epigenetic and regulatory information are needed to more fully determine how stock phenotypic diversity is regulated.

## Background

Managing genetic diversity is critical for the success and sustainability of breeding programs of domesticated organisms. Retaining enough genetic diversity while simultaneously applying selection for desirable traits is a balance that ensures future success through reservoirs of standing variation and minimizes the likelihood of genetic bottlenecks [1–3]. Honey bees (*Apis mellifera*) are the predominant managed insect pollinator for a majority of food crop species and depend on high genetic diversity within colonies and between populations. High intracolony diversity in honey bees promotes fitness [4–7], productivity [8, 9] and colony survival [10]. At the population level, low genetic diversity may contribute to the long-term declines of managed colony numbers, and to the more recent trends of high annual mortality and poor health of colonies [5, 11, 12].

There are currently a number of honey bee stocks (supposedly isolated populations bred for desirable traits) produced by queen breeders for widespread use across the United States. The genetic background favored by the majority of U.S. beekeepers is derived primarily from the subspecies *A. m. ligustica*; such bees are generally referred to as Italian honey bees. Over the years, Italian stocks have been selected primarily for honey production, colony size, and body color [13]. Other widely used stocks are derived from the related subspecies *A. m. carnica*, known as Carniolan bees. The majority of queens that are commercially produced in the U.S. originate from a relatively small number of queen producers in California, Hawaii and the southern U.S. It has been suggested by previous work that a combined 500 breeder queens across operations are used to rear over a million daughter queens that are then distributed throughout the U.S. [13]. While these do not all represent genetically distinct lines, the varied sources contribute to genetic diversity among stocks. In addition, there are also less frequently used stocks developed for more specialized traits or as endpoints of research-based breeding programs such as Russian, Minnesota Hygienic, Pol-line, Mite-biter and Saskatraz [14–19]. The breeding schemes for these stocks vary. For example, Russian bees are bred in a highly structured closed breeding program, consisting of 16 distinct lines and directed crosses (via open mating) among those lines [20]. The Pol-line breeding program is also a closed system, propagating 12–16 queen lines annually, however mating occurs via instrumental insemination of pooled drone semen [21]. In contrast, Minnesota Hygienic bees are propagated based on colony phenotype, independently by commercial beekeepers [14]. The variance in breeding and population structure among managed honey bee colonies has, as of yet, lacked a comprehensive high resolution understanding of the genetic diversity and structure in managed U.S. honey bee populations. A population genetic approach to the analysis of honey bee genetic variation could prove useful by identifying regions that have undergone selection through breeding efforts which can be used as a basis for novel breeding programs. Previous work has provided genomic information about aggressive behavior, swarming, and Africanization in various honey bee populations globally [22–25]. Several studies have described the overall diversification of U.S. honey bees, with a focus on the origins of managed and feral populations [10, 13, 26–30]. A more comprehensive understanding of the genetic differentiation among commercially relevant stocks, however, is an important first step toward efforts to begin implementing genomic-based marker-assisted selection to rapidly and efficiently improve honey bee breeding programs.

We investigated the current patterns of genetic diversity of eight U.S. honey bee stocks using whole genome sequencing of pooled individuals (Pool-seq) to determine genome-wide allele frequencies. This cost-effective approach facilitates the sampling of a large number of individuals while effectively calling conserved alleles segregating within the population to better understand the wider population genetic structure [31]. We characterize the genetic diversity found within and between these stocks and identify single nucleotide polymorphisms (SNPs) that may be useful in stock identification. Furthermore, we used this information along with population demographic data to discover genomic regions likely under selection for enhanced resistance to pathogens and parasites. The overall aim was to provide an initial genetic screen to strengthen future efforts toward incorporating the use of marker-assisted selection in honey bee breeding, especially for traits that are challenging to phenotype.

## Results

We evaluated seven U.S. honey bee stocks collected in 2016 and one collected in 2019 (Hilo). Four of the stocks are widely used in the beekeeping industry including three Italian (designated as Italian1, Italian2 and Italian3) and one Carniolan, representing some of the major bee breeding programs in North America. The remaining four stocks (Minnesota Hygienic, Pol-line, Hilo and Russian), are products of specialized breeding efforts. Minnesota Hygienic bees were originally derived from Italian bees and were selected for enhanced expression of hygienic behavior via a freeze-killed brood assay [32]. This trait affords resistance to several pathogens and, to a lesser extent, the parasitic mite *Varroa destructor* [32–34]. Proteomic and QTL analysis have identified several promising candidate genomic regions that may assist in future biomarker identification related to general hygienic behavior [35–37]. Pol-line honey bees were derived from Italian bees with high *Varroa* resistance largely derived from the expression of the ‘*Varroa*-sensitive hygiene’ (VSH) trait [38]. Several studies have attempted to profile the genetic and biochemical mechanisms regulating hygienic behavior [39, 40], though the precise regulatory mechanisms managing this trait are still unknown. Hilo stock is from an ongoing breeding program that combines VSH behavior from Pol-line with Italian stocks for improved honey production and colony size. Russian bees were selected for low mite population growth of *Varroa* [19, 41], and display VSH as one mechanism of resistance [19, 42]. We considered stock-specific differences individually in our assessments of variation. However, in instances where we examined variation derived from targeted breeding efforts in the research stocks, we combined all three Italian stocks to form a common background under the assumption that their combined profile is representative of the broader genetic diversity of managed Italian bees across the U.S.

### Within-stock genome-wide variation

**Fig. 1:**
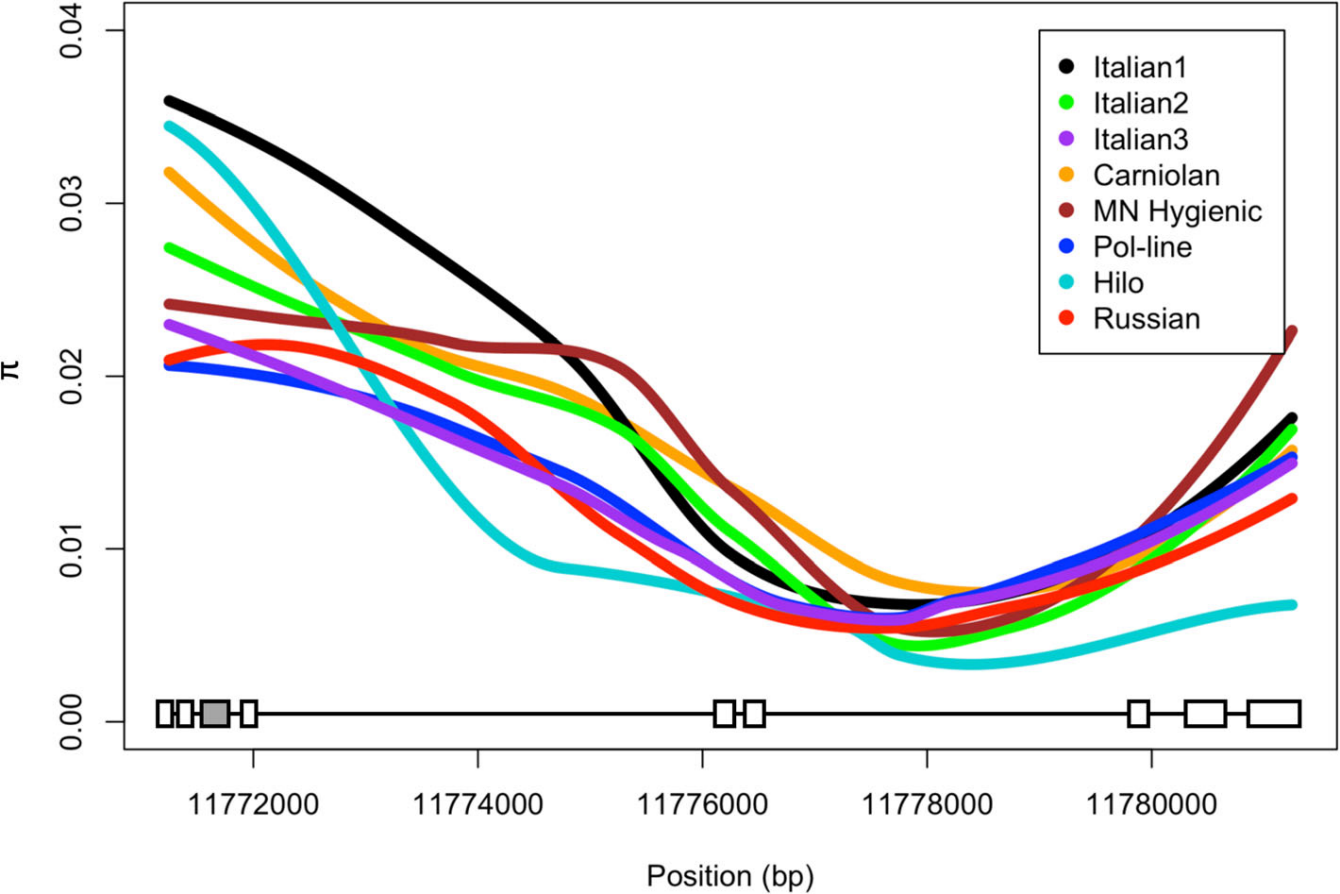
Smoothed fit of nucleotide diversity (π) across the complementary sex determiner, *csd*, locus estimated over 250 nonoverlapping windows for each stock. *Csd* exons are depicted across the base of the figure. Exon 7, the hypervariable region, is depicted in grey

We found ~ 1.2–1.8 million polymorphic SNPs within each stock (Table 1). Estimates of π and θ averaged over the whole genome were lowest in Italian3 (π = 4.226E-03; θ = 4.306E-03) followed closely by Pol-line (π =4.361E-03; θ = 4.377E-03). Highest estimates of π and θ diverged, with Italian2 having the highest estimate of π (5.084E-03) and Carniolan having the highest estimate of θ (5.099E-03, Table 1). Estimates of π over the *csd* locus suggest that Italian1 is the most genetically diverse across this region, while the Hilo is the least genetically diverse (Fig. 1, Supplemental Table 2).

**Table 1:** Pi, Theta, and the average total number of SNPs measured among the eight honey bee stocks

Stock	π	휃	Average Total No. SNPs
Italian 1	4.998E-03	5.034E-03	1,458,101
Italian 2	5.084E-03	4.819E-03	1,465,571
Italian 3	4.226E-03	4.306E-03	1,439,926
Carniolan	4.712E-03	5.099E-03	1,422,100
Minnesota Hygienic	5.073E-03	5.035E-03	1,538,237
Pol-line	4.361E-03	4.377E-03	1,423,510
Hilo	4.848E-03	4.840E-03	1,447,778
Russian	4.700E-03	4.681E-03	1,423,499

**Fig. 2:**
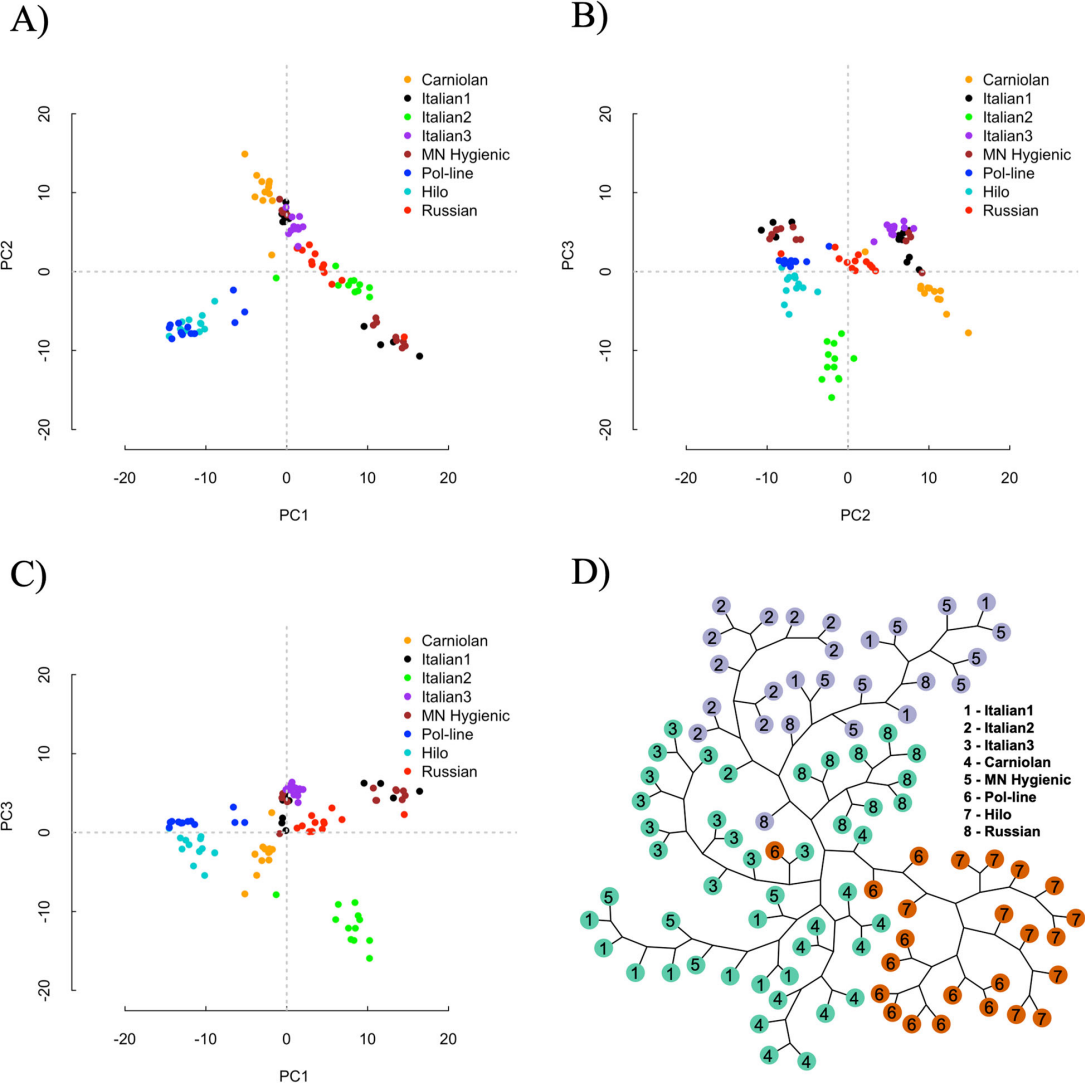
Principal component analysis of allele frequencies by principal components (PC) and k-nearest neighbor (KNN) analysis of the eight sequenced honey bee stocks demonstrating the distribution of genetic relatedness. PC 1 and 2 (**a**); PC 2 and 3 (**b**), PC 1 and 3 (**c**), and KNN (**d**)

The covariation of allele frequencies of a subset of 477,735 SNPs, where at least one variant existed among the eight stocks at a frequency > 0.1, were used to examine the genetic structure among stocks. Two analyses were used for this process: a principal component analysis to dimensionally reduce the genetic variation per sample, and a k-nearest neighbor (KNN) clustering analysis to identify broad genetic clusters among the eight stocks. The first principal component (PC1) accounted for 5.1% of the variation in allele frequency data, while PC2 and PC3 accounted for 3.9 and 2.6% of the variation, respectively. Broadly, PC1 appeared to differentiate among the Carniolan-Italian spectrum of genetic diversity while PC2 primarily differentiated Pol-line and Hilo stocks (Fig. 2a). PC3 differentiated the Italian2, Hilo, and Carniolan stocks (Fig. 2b-c). The KNN analysis identified three genetic subgroups among the eight sequenced stocks. Stocks within these subgroups were likely experiencing continued gene flow or lacking the generational time to have diverged. The KNN analysis further highlighted Pol-line and Hilo as highly differentiated from other commercial stocks (Fig. 2d).

### Population differentiation

**Fig. 3:**
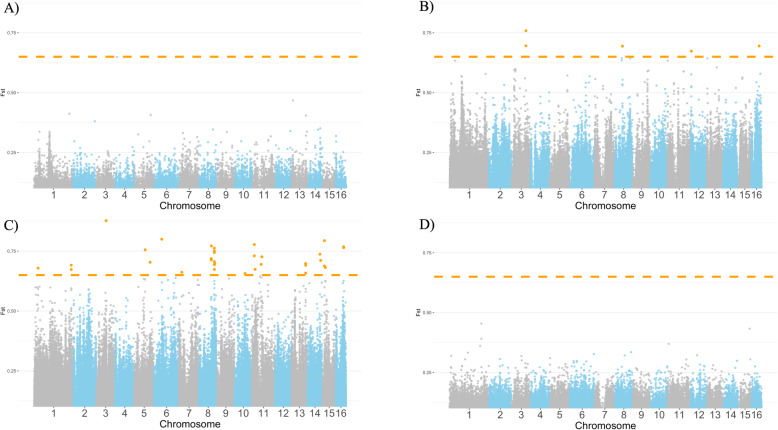
Pair-wise F_ST_ measures between combined Italian stocks and **a**) Minnesota, **b**) Pol-line, **c**) Hilo, and **d**) Russian stocks bred for resistance traits. F_ST_ > 0.65, highlighted in orange, depicts significant fixed differentiation between the comparisons

In order to investigate stock differentiation between populations from different genetic backgrounds, we estimated pairwise F_ST_ at each polymorphic site. Average genome-wide measures of F_ST_ found the greatest differentiation between Minnesota Hygienic and Hilo stocks (F_ST_ = 0.031), while Russian and Minnesota Hygienic stocks were all similarly less differentiated from the generalized Italian stock (F_ST_ = 0.011, Table 2). Pairwise F_ST_ comparisons between the generalized Italian stock and those selected for parasite and pathogen resistance (i.e., Minnesota Hygienic, Pol-line, Hilo, and Russian stocks) found that only Pol-line stock had a wider range of F_ST_ demonstrating greater differentiation from the Italian stock (Fig. 3). We found a total of 422 candidate SNPs that may be used to differentiate pairs of stocks, based on pair-wise comparisons for SNPs with a high degree of fixed differentiation (F_ST_ > 0.65) (Table 2) [37, 43].

**Table 2:**
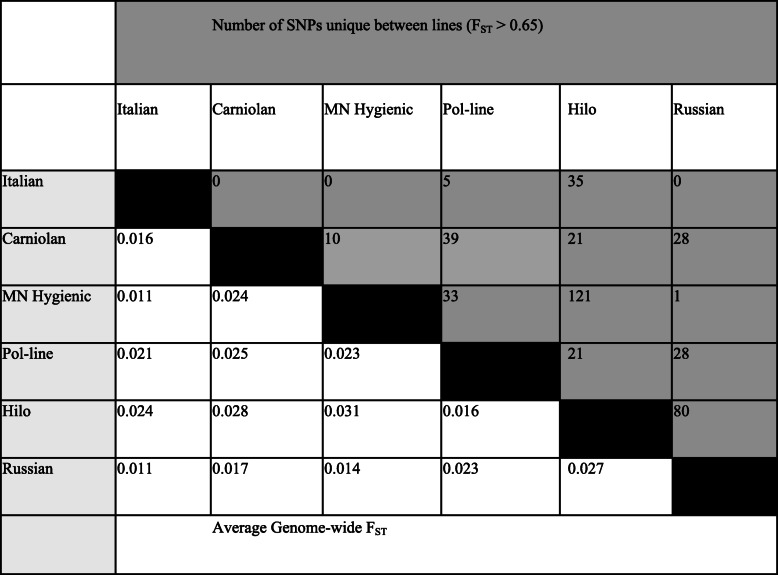
Stock specific measures of genetic diversity within honey bees. Shaded values indicate the number of highly differentiated pair-wise (F_ST_ > 0.65) SNPs, while unshaded cells show the average genome-wide F_ST_ values

To establish unique stock-specific SNPs among all of the stocks examined as possible candidates for stock identification, we analyzed SNPs with the top 1.0 and 0.1% F_ST_ values and extracted only the unique SNPs. Within the top 1.0% F_ST_ value SNPs we found a range of 5–405 SNPs that are putatively stock-specific (Table 3). However, there were 0–12 stock-specific SNPs when only the top 0.1% of FST value SNPs were considered. A full list of the stock-specific SNPs in the top 0.1% can be found in Supplemental Table 3.

**Table 3:** Number of Stock-Specific SNPs among the Top 1.0 and 0.1% of F_ST_ values

Stock	Unique SNPs Among the Top 1.0% of F_**ST**_ SNPs	Unique SNPs Among the Top 0.1% of F_**ST**_ SNPs
Italian1	5	0
Italian2	275	10
Italian3	91	9
All Italian	0	0
Carniolan	127	4
Minnesota Hygienic	106	1
Pol-line	157	12
Hilo	405	1
Russian	26	1

### Genomic regions under selection

A total of 46 haplotype blocks containing the most significant Composite Selection Signal (CSS) scores (top 0.1%) among generalized Italian, Minnesota Hygienic, Pol-line, Hilo, and Russian stocks with more than five SNPs were identified. Several regions demonstrated overlap with regions previously associated with general hygienic behavior or VSH (Table 4). A full list of the top 0.1% CSS SNPs can be found in the Supplemental Table 4 and the distribution of CSS scores can be found in Supplemental Figure 1. In the most significant CSS score regions, gene enrichment was found in categories of *integral component of membrane* (GO:0016021); and UniProtKB keywords *transmembrane, transmembrane helix*, and *membrane associated clustering*.

**Table 4:** CSS regions identified to be under high positive selective pressure among Minnesota Hygienic, Pol-line, Hilo, and Russian stocks putatively for general hygienic behavior or VSH (Tsuruda et al. 2012, Splotter et al. 2012). Position information of enriched blocks, genes and overlap with previously identified QTLs are shown

Chr	Starts	Ends	SNPcount	Gene	Previously Identified Hygienic Association
LG1	2,345,989	2,346,935	6	LOC724238	
LG1	2,909,659	2,911,035	6	LOC410758	
LG1	3,761,417	3,762,423	6		Spötter et al. 2012
LG1	7,819,348	7,820,225	7		
LG1	8,889,621	8,890,832	7	LOC410732	
LG1	8,891,866	8,892,300	6	LOC410732	
LG1	8,927,676	8,928,305	6		
LG1	9,466,743	9,467,416	6	LOC409608	
LG1	9,491,950	9,492,350	10	LOC100578747, LOC409608	
LG1	21,158,620	21,159,496	6	LOC413968	
LG2	6,660,797	6,661,595	8	LOC725588	
LG2	9,716,385	9,717,106	11	LOC551772	Spötter et al. 2012
LG3	9,550,121	9,550,612	8		
LG3	10,115,687	10,116,744	10	LOC413829	Spötter et al. 2012
LG3	10,530,176	10,530,589	10	LOC410926	Spötter et al. 2012
LG3	12,691,622	12,696,809	9	LOC552149	
LG4	13,011,199	13,011,553	8	LOC551628	
LG5	6,479,299	6,479,897	8	LOC113218552, LOC411079	
LG6	13,890,152	13,890,546	7	LOC102656661	
LG6	13,905,063	13,906,782	6	LOC102656661	
LG6	14,675,405	14,675,960	6	LOC107964545	
LG7	674,945	675,266	7	LOC412896	
LG7	12,686,713	12,687,509	10	LOC408915	
LG8	2,114,043	2,120,983	6	LOC102654371	
LG8	2,188,402	2,193,281	6	crh-BP	
LG8	10,973,296	10,974,126	6		Spötter et al. 2012
LG9	10,466,886	10,467,800	13		Tsuruda et al. 2012
LG10	6,334,326	6,335,519	10		
LG10	10,804,935	10,805,598	6	LOC100577980, LOC411288	
LG11	710,227	727,890	6		
LG11	3,445,285	3,456,385	7		
LG11	11,299,503	11,300,480	6	LOC724460, LOC726180	
LG11	14,537,227	14,539,063	11	LOC102653678	
LG12	1,549,766	1,551,073	6		
LG12	2,294,313	2,298,222	7	LOC411813	
LG12	3,715,250	3,715,959	7	LOC100578102	
LG12	5,861,319	5,861,711	7	LOC724292	
LG13	849,828	851,798	6		
LG14	7,837,851	7,838,138	6	LOC410515	
LG14	8,891,482	8,892,699	10	LOC410535	
LG14	9,640,842	9,641,372	10		
LG16	261,473	262,437	6	LOC107965756	
LG16	874,080	874,571	6		
LG16	2,345,631	2,346,711	11	LOC724468	
LG16	2,634,030	2,634,557	8	LOC413789	
LG16	4,418,236	4,419,113	6		

## Discussion

This study represents the first genetic characterization of U.S. honey bee stocks. We show that within-stock genetic diversity is limited. The genetic variation that exists was largely shared among stocks with few markers found to be unique to each. We show that Pol-line and Hilo are the most distinct among the stocks, with the largest number of differentiated markers. These results confirm previous work indicating a high degree of admixture among honey bee populations in North America [44] and that targeted breeding for very specific traits can quickly differentiate populations.

### Genetic diversity metrics: within stocks

We found relatively similar, low levels of within-population genetic diversity measured by π and θ across the genome for the eight stocks. It should be noted that a few factors may potentially be influencing our findings such as the matrilineal bias influenced by honey bee multiple mating, our pooling approach, and potential non-random sampling of individuals by bee breeders. However, the differences in within-stock variation examined among stocks provides an informative, relative measure of genetic diversity. In regard to possible over-sampling of related individuals within a population, we do not observe any distinguishing effects between the commercial and highly controlled Hilo, Pol-line, and Russian populations. This suggests that within sample relationships may not be contributing significantly to the reduced genetic diversity.

Admixture appears to cause commercially managed honey bees to have greater genetic diversity than their progenitor populations [45]; we thus expected the commercial Italian and Carniolan stocks to have the highest levels of π and θ when compared to the specialized research-based stocks. However, we found inconsistent levels of genetic diversity both in the commercial and research-based stocks. Although our genome-wide estimates of π and θ show low genetic diversity within stocks, analysis of the *csd* locus provided greater resolution of recent population changes. Honey bees possess a single-locus sex determination system where homozygosity at the *csd* locus results in functionally non-viable diploid males [46]. Genetic variation at this locus has been demonstrated to be critical for colony health because of its effect on brood viability [47, 48], and has been used to calculate estimates of mating population size and broad genetic diversity [23, 47]. It is interesting to note that although the Hilo stock exhibited moderately high genome-wide π and 휃 estimates, it had the lowest such estimates at the *csd* locus. This suggests a disconnect between genomic level nucleotide diversity and that of the *csd* locus, which is possible in honey bees given their high rate of recombination and low levels of linkage-disequilibrium [46]. Further exploration of this will be informative for adequate determination and mitigation of inbreeding depression in honey bee breeding operations.

### Population variation: among stocks

Differences in genetic variation among stocks identified three general clusters which principally grouped as Hilo-Pol-line, Italian1-Italian3-Russian-Carniolan, and Italian2-Minnesota Hygienic. This provides further evidence of the genetic differentiation of Hilo and Pol-line from other stocks. We posit this was largely driven by a combination of continued selective pressure for a specific, shared mite-resistant phenotype (VSH) along with relative reproductive isolation. Interestingly, though Hilo and Italian2 stocks have some shared ancestry (4 of the 12 Hilo colonies had an Italian2 maternal lineage), Hilo primarily clusters with Pol-line. This may indicate a greater contribution of the Pol-line ancestry to Hilo, with F_ST_ estimates suggesting that Hilo is more differentiated from the combined Italian stocks (F_ST_ = 0.024) than they are from Pol-line (F_ST_ = 0.016). Additionally, these findings may reflect a paternal effect as the relationship between these stocks is through some sharing of patrilineal lineages. Other stocks that were either selected for hygienic behavior (Minnesota Hygienic) or express it at a high level without targeted selection for the trait (Russian) did not show a similar degree of differentiation from the common Italian background, potentially highlighting that VSH behavior has at least some partially unique genetic features. This may be a function of increased independent selection for hygienic behavior by bee breeders [49] in the Italian based stocks and the high likelihood of introgression from surrounding populations. This may also explain the distribution of the Russian and Minnesota Hygienic stocks across two genetic clusters determined through the KNN analysis as a result of shared selection pressure among operations.

The majority of SNPs identified in this study did not demonstrate a significant degree of differentiation between stocks. Hilo and Pol-line had the most SNPs at the 1.0% (*n* = 405) and 0.1% (*n* = 12) cutoffs, respectively. This further highlights the genetic differentiation of these populations compared to other stocks, likely in response to targeted selection focused largely on one trait (VSH). The strong genetic similarity among Italian, Russian, Minnesota Hygienic, and Carniolan stocks yielded few unique SNPs that could effectively segregate the stocks at the highest F_ST_. Interestingly, Carniolan and Russian stocks had very few SNPs differentiating them from the generalized Italian stock despite the controlled nature of their breeding programs. This supports the case that introgression from external Italian honey bee populations exists either from their population of origin (e.g. [50]) or after their introduction in the U.S. For example, while Russian honey bee stock is produced as part of a closed network of breeders [44], queens are produced via natural mating. As such, there is an increased likelihood of admixture, as has been seen in recent stock assessment [51]. Despite these populations appearing relatively genetically similar to one another, there remain significant and observable phenotypic differences between stocks. The underlying variation driving the phenotypic differences may be caused by mutations not detected in this study such as indels, inversions, or low frequency variation among polygenic traits. However, differences in and between populations were still detected despite the conservative nature of our analyses. Furthermore, differences in gene regulatory or epigenetic profiles may also be influencing differences between stocks and are potential avenues for further investigation. The findings summarized above provide several lines of evidence consistent with the genetic effects of selective breeding in combination with high levels of admixture within U.S. honey bee populations.

### Signatures of selection and identification of candidate genes

CSS scores improve the power to detect and resolve selection signals and localize candidate regions involved in traits experiencing selection pressure. Regions under selection shared by Minnesota Hygienic, Pol-line, Hilo, and Russian stocks provide actionable targets for future research and breeding. Our annotation using haplotype blocks identified 46 of the 58,333 [23] that were shared among the four stocks with a strong signal of selection providing evidence of a common selection signal among stocks associated with mite- and disease-resistance traits. However, there remains an unlikely possibility that a CSS signal may arise of a specific region that is highly selected in only the Italian stocks and not of the other populations. Though we feel that it is more likely that the research stocks are arriving at a shared resistance given the intent of their respective programs. Overlap between our CSS signal and previous QTLs associated with hygienic behavior and social immunity was found across LG 1, 3, 7, 8, and 9 [37, 52, 53]. Our findings further support that the active selection for behavior conferring improved resistance to parasites and pathogens, along with the increased fitness of these traits, may be shared among these lines. Further investigations to determine the functional mechanisms regulating social immunity will aid in the development of molecular tools to apply for more full integration of resistance traits among honey bee breeding programs and populations. Certainly, the successful use of proteomic markers for the selection of hygienic behavior highlights the value for the integration of functional genomics in work related to genomic markers for selection [35, 54, 55]. Given the complex regulatory network influencing disease and parasite resistance traits, significant work remains before marker assisted selection can be employed on a large-scale and breeding efforts focusing on resistance phenotypes must continue to be supported.

## Conclusions

This study categorizes the underlying genetic variation that exists in common honey bee stocks through the use of genomics and population genetic approaches. The findings suggest the need for future efforts to integrate additional forms of data including gene expression, epigenetic, and regulatory information to provide a more complete understanding of the mechanisms regulating stock phenotypic diversity. Validation of QTLs for complex traits associated with disease and parasite resistance among honey bee populations has been extremely difficult to achieve [42]. Understanding of which genomic regions are shared or are particularly differentiated is an important step forward. Through the incorporation of the results presented here and future efforts into data repository resources such as Hymenopteramine [56], beekeepers and honey bee researchers can begin to work towards the development of marker-assisted selection to “build a better bee.”

## Methods

### Sample preparation and sequencing

Pool-seq was used to widely sample each population and identify conserved patterns of genetic variation within each of the 8 sampled stocks. To sample, 96 worker bees were collected from 12 breeder colonies of each stock. The Carniolan, Minnesota Hygienic and three Italian stocks were randomly sampled by the bee breeders to be sent for sequencing. Given that Hilo, Pol-line, and Russian stocks are either maintained by the USDA-ARS Baton Rouge research unit or close collaborators, they were carefully sampled to ensure independent sampling within each of the populations so as to minimize relatedness between colonies. Colonies from the Russian stock were selected from 12 of the 18 breeding lines, Pol-line colonies represented 12 different lines and Hilo represents 10 different lines. Eight pools of 12 heads per colony were prepared by first excising the heads using sterile surgical scalpels. The compound eye lenses were then removed to limit introduction of magnesium residues into downstream reactions [57]. Samples were held at -20 °C prior to DNA extraction. Each pool was first homogenized using Omni BeadRuptor™ – Elite 2.0 mL Soft Tissue Kit vials pre-fitted with 1.4 mm ceramic disruption beads. Final DNA extraction was completed using either Qiagen DNeasy Blood and Tissue Kit (Qiagen USA, CAT#69506) following manufacturer recommended protocol for single tube extraction or Sbeadex^Ⓡ^ purification kits (Biosearch Technologies UK, CAT#NAP41450). The extraction method for each sample is listed in Supplementary File Table 1. Final quality check was performed using Quant-iT™ PicoGreen™ dsDNA assay kit (Invitrogen USA, CAT#P11496).

Sequencing was performed at the Institute for Genome Science at the University of Maryland School of Medicine using the Illumina HiSeq4000 150 paired-end reads. The 96 pools of samples were spread across eight lanes for a mean coverage depth of 30x per sample. Raw sequence files were trimmed using Trim Galore v0.6.0 and then aligned to the honey bee reference genome (Amel_HAv3.1) using bwa v0.7.16a to generate bam files [58]. Duplicates were marked using picard tools’s MarkDuplicates (http://broadinstitute.github.io/picard/) on the trimmed fastq files followed by SortSam to sort reads. Sorted reads were then processed by GATK’s BaseRecalibrator to correct for patterns of systematic errors in base quality scores using base settings [59–61].

### Variant calling

SNPs were derived for our data set by using using Lofreq’s “call” function [62] which produces a VCF file used in the subsequent downstream analysis. Allele frequency information was extracted and visualized from Lofreq produced VCF files using SNPRelate [63]. Variants were called for each population separately, filtered by the above quality measures.

### Summary of genetic variation

A joined set of genetic variants with at least one overlapping SNP was used to conduct a principal component analysis (PCA) to summarize the covariation in allele frequencies between samples. The code used to generate the overlapping SNP set is provided in Supplemental File 1. An iterative k-means hierarchical analysis was applied to the resulting principal components (PC) to identify the optimal number of genetic clusters in our data set agnostic of population membership [64]. The Akaike Information Criterion (AIC) was used on a range of 1–10 possible clusters and the optimal number of clusters was selected using the elbow method.

### Estimation of population genetic parameters and stock specific SNPs

Genome-wide patterns of differentiation between the sequenced stocks was estimated with the population genetic parameters pi (π), theta (θ), and F_ST_ [65–67]. For the within and between pairwise comparisons of the stocks, the software packages Popoolation was used to estimate π and θ [68] and Popoolation2 was used to estimate F_ST_ [69]. Both these packages used mpileup files generated though samtools from the recalibrated bam files. In estimates of π and θ, we adjusted the run parameters of Popoolation and Popoolation2 to include a minimum coverage of 20 reads, a minimum count of 10 reads, and a minimum quality threshold of 20. We also used a max-coverage threshold of no more than 2% of the total number of reads. These filters frame assured the consideration of the most conservative and consistent SNPs among our data set. A generalized Italian population was generated by merging the commercial stocks advertised as Italian in origin. SNPs were considered to have a high degree of fixation difference between pairwise comparisons of generalized Italians, Pol-line, Hilo, Russian, and Minnesota Hygienic stocks when F_ST_ was > 0.65 [43].

Stock specific SNPs were identified using the top 1.0 and 0.1% F_ST_ values from each of the pairwise comparisons of stocks. These most highly differentiated SNPs were then compared among the six stocks (Carniolan, Minnesota Hygienic, Pol-line, Hilo, Russian, and generalized Italian) to identify SNPs unique to that stock among these top candidates.

### Composite selection signal score to identify signals of selection

A composite selection signal (CSS) score was used to refine and detect signatures of selection [70]. This method unifies multiple estimates of population differentiation measurements in order to capture highly differentiated loci and their respective genetic variants being enriched within the population. Briefly, a fractional rank of the F_ST_ calculations for Minnesota Hygienic-Italian, Pol-line-Italian, Hilo-Italian, and Russian-Italian comparisons were calculated and used to derive a Z-score. The Z-score was averaged for each corresponding SNP, compared to a standard distribution to derive a *P* value, and transformed using a -Log_10_(*P* value) representing the CSS [70]. CSS outliers were identified as having a CSS score in the top 0.1% of the distribution. To assist in functional annotation, we capitalized on the small span of linkage-disequilibrium across the honey bee genome. Candidate SNPs from our CSS values were localized to previously described haplotype blocks [23]. These haplotype blocks are genomic spans with conservative estimates of linkage-disequilibrium derived across three genetically distinct populations. We considered only haplotype blocks found to have > 5 SNPs/block. The lengths of remaining haplotype blocks ranged from 287 to 17,663 base pairs. After overlap with our CSS results we arrived at 58,333 haplotype blocks with at least one SNP from our CSS analysis. This subset was used to identify genes located within genomic blocks with high CSS scores. We used DAVID functional annotation tool v6.8 and HymenopteraMine to identify clusters of gene ontology terms enriched in highly selected regions [56, 71, 72] with a minimum enrichment score of 1.3 for gene clusters and the Benjamini corrected *P*-value as the cutoff using the *Apis mellifera* background. All statistical analyses were performed with R v3.6.0 [73].

## Data Availability

Sequencing data presented in this study has been deposited at NCBI under the accession PRJNA605407.

## Additional ffiles

**Additional file 1 : Supplemental Table 1**. DNA sampling method. **Additional file 2 : Supplemental Table 2**. Average π across the *csd* locus for all sequenced stocks. **Additional file 3 : Supplemental Table 3**. Stock-Specific SNPs and F_ST_ values for the top 1% of F_ST._ **Additional file 4 : Supplemental Table 4**. A full list of the top 0.1% significant CSS regions. **Additional file 5 : Supplemental File 1**. R code used to find overlapping SNPs for PCA analysis. **Additional file 6 : Supplemental Figure 1**. Distribution of the CSS score.

## Abbreviations

SNP: Single nucleotide polymorphism
CSS: Composite selection score
QTL: Quantitative trait loci
VSH: Varroa sensitive hygiene
KNN: k-nearest neighbors
AIC: Akaike information criterion
