# Supplementary figures and images for: Genome-wide patterns of differentiation within and among U.S. commercial honey bee stocks
Source: BMC Genomics. 2020 Oct 8;21:704. doi: 10.1186/s12864-020-07111-x (PMC7545854; doi:10.1186/s12864-020-07111-x)

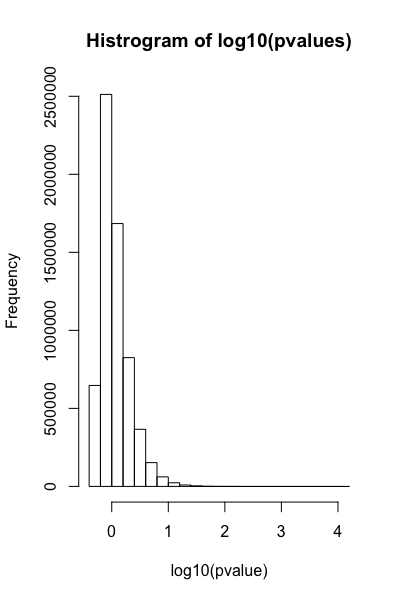

Supplement: Supplementary file 6 — Additional file 6 : Supplemental Figure 1. Distribution of the CSS score. [file 12864_2020_7111_MOESM6_ESM.tiff]
